# Inhibition of tumor progression during allergic airway inflammation in a murine model: significant role of TGF-β

**DOI:** 10.1007/s00262-015-1722-4

**Published:** 2015-06-16

**Authors:** Belen Tirado-Rodriguez, Guillermina Baay-Guzman, Rogelio Hernandez-Pando, Gabriela Antonio-Andres, Mario I. Vega, Leticia Rocha-Zavaleta, Laura C. Bonifaz, Sara Huerta-Yepez

**Affiliations:** Unidad de Investigación en Enfermedades Oncológicas, Hospital Infantil de México Federico Gómez, Dr. Márquez No 262, Col. Doctores, Delegación Cuauhtémoc, C.P. 06720 Mexico City, Mexico; Programa de Doctorado en Ciencias Biomédicas, Facultad de Medicina, Universidad Nacional Autónoma de México (UNAM), Mexico City, Mexico; Experimental Pathology Section, Department of Pathology, National Institute of Medical Science and Nutrition, Salvador Zubiran (INCNSZ), Mexico City, Mexico; Oncology Research Unit, Oncology Hospital, Centro Medico Nacional Siglo XXI, Instituto Mexicano del Seguro Social (IMSS), Mexico City, Mexico; Departamento de Biologia Molecular y Biotecnología, Instituto de Investigaciones Biomédicas, Universidad Nacional Autónoma de México (UNAM), Mexico City, Mexico; Unidad e Investigación Médica en Inmunoquímica, Centro Médico Nacional Siglo XXI, Instituto Mexicano del Seguro Social (IMSS), Av. Cuauhtémoc 330, Col. Doctores, Delegación Cuauhtémoc, C.P. 06720 Mexico City, Mexico

**Keywords:** Allergic airway inflammation, Breast cancer, TGF-β, Apoptosis, Allergo-oncology

## Abstract

**Introduction:**

TGF-β is an important mediator of pulmonary allergic inflammation, and it has been recently reported to be a potential inhibitor of lung tumor progression. The correlation between cancer and allergic inflammatory diseases remains controversial. Thus, the aim of the present study was to evaluate the effects of pulmonary allergic inflammation and in particular the role of TGF-β on cancer progression.

**Methods:**

Cancer cells were implanted in a BALB/c mice model of allergic airway inflammation, and tumor growth was measured. Apoptosis was evaluated by TUNEL assay, and TGF-β was measured by ELISA. Expression of proliferating cell nuclear antigen, TGF-β, TGF-β receptors I and II, phospho-Smad2 and phospho-Smad4 was evaluated by immunohistochemistry and quantified using digital pathology. The effect of a TGF-β activity inhibitor and recombinant TGF-β on tumor growth was analyzed. The effect of exogenous TGF-β on cell proliferation and apoptosis was evaluated in vitro.

**Results:**

Mice with allergic airway inflammation exhibited decreased tumor volumes due to cell proliferation inhibition and increased apoptosis. TGF-β was increased in the sera and tumor tissues of allergic mice. TGF-β activity inhibition increased tumor progression in allergic mice by enhancing proliferation and decreasing apoptosis of tumor cells. The administration of TGF-β resulted in reduced tumor growth.

**Conclusion:**

This study is the first to establish an inverse relationship between allergic airway inflammation and tumor progression. This effect appears to be mediated by TGF-β, which is overexpressed in tumor cells during pulmonary allergic inflammation. This study indicates that TGF-β is a potential target for antitumor therapy.

**Electronic supplementary material:**

The online version of this article (doi:10.1007/s00262-015-1722-4) contains supplementary material, which is available to authorized users.

## Introduction

Based on epidemiological data, several research groups have found an inverse correlation between cancer and allergic inflammatory diseases [1–3]. Asthma is a chronic airway disease in which inflammatory processes and bronchial hyperreactivity lead to a reversible bronchial airway obstruction that is characterized by a Th2 response [4–7]. One of the most important cytokines known to mediate allergic reactions is transforming growth factor β (TGF-β), which is an important fibrogenic factor and immune modulator that may play a relevant role in generating structural airway changes in asthma patients [8–11]. In vivo and in vitro studies have demonstrated that TGF-β acts as a pro-inflammatory cytokine, initiating and enhancing inflammation during airway immune responses. This cytokine is also involved in airway remodeling, particularly in the development of sub-epithelial fibrosis. TGF-β is produced by inflammatory cells of the bronchial mucosa and by bronchial wall cells, such as fibroblasts, epithelial cells, endothelial cells and smooth muscle cells [8, 12, 13]. A significant increase in TGF-β has been observed in the bronchial lavage of asthma patients, suggesting that the level of this cytokine is directly correlated with the severity of disease [9, 12, 14].

The possibility that a biological relationship exists between allergies and cancer has intrigued researchers and health professionals. Although several groups have explored this possible relationship, no consensus has been reached [15]. Despite the conflicting evidence, currently, no studies have evaluated the functional effects of allergic airway inflammation that is characteristic of asthma on cancer progression in experimental models.

Recently, a mice model was developed in which pulmonary allergic inflammation is triggered by exposure to ovalbumin (OVA) and cancer is induced with urethane. The results demonstrated that the allergic status had no influence on tumor development. Interestingly, a recent study using mice overexpressing Smad7, an inhibitor of the TGF-β signaling cascade in Clara lung cells, revealed that there is a significant increase in urethane-induced lung cancer progression that is associated with TGF-β inhibition in this organ [16]. This observation emphasizes the importance of TGF-β in suppressing tumor progression, particularly in lung cancer induced by a chemical compound. This finding is highly relevant because it suggests that TGF-β may play an important role in inhibiting tumor progression, which is in contrast to most of the previously published studies suggesting that this cytokine promotes the progression of different types of cancer [17, 18]. Thus, the aim of the present study was to evaluate the effect of allergic inflammation, particularly the role of TGF-β, in the development of cancer using a mice model of pulmonary allergic inflammation followed by the transplantation of syngeneic breast cancer cells. In our model, allergic airway inflammation inhibited cancer progression. A concomitant increase in systemic and intratumoral TGF-β levels was observed in allergic mice, along with an increase in apoptotic cells. The inhibition of TGF-β activity induced tumor progression. Our results support the hypothesis that there is a negative correlation between allergy and cancer and also suggest that TGF-β may be a mediator of this effect.

## Methods

### Reagents and antibodies

Ovalbumin (OVA) grade V was obtained from Sigma-Aldrich (St. Louis, MO, USA). Aluminum hydroxide (alum) was obtained from Pierce Biotechnology (Rockford, IL, USA). Normal rabbit IgG was used as an isotype control (IC), and anti-TGF-β, antiproliferating cell nuclear antigen (PCNA), anti-TGF-β receptor I (TβRI), anti-TGF-β receptor II (TβRII), and biotin-conjugated antigoat antibodies were purchased from Santa Cruz Biotechnology (Santa Cruz, CA, USA). Rabbit anti-phospho-Smad2 (Ser 465/467) and anti-phospho-Smad4 antibodies were purchased from Cell Signaling Technology (Danvers, MA, USA). The TβRI inhibitor SB431542 (4-[4-(1,3-benzodioxole-5-yl)-5-(2-pyridinyl)-1H-imidazole-2-yl]-benzamide; 4-[4-(3,4-methylenedioxyfenyl-5-(2pyridyl)-1H-imidazole-2yl]-benzamide; 4-(5-benzol-[1, 3] dioxol-5-yl-4-pyridine-2-yl-1H-imidazole-2-yl)-hydrated benzamide), which inhibits TGF-β1 activity [19], were obtained from Sigma-Aldrich. Recombinant TGF-β was obtained from R&D (R&D, Minneapolis, MN, USA), and a cytometric bead array (CBA)-based mouse Th1/Th2/Th17 cytokine kit was purchased from BD (BD, Franklin Lakes, NJ, USA).

### Animals and cells

Female BALB/c mice (6- to 8-week-old) were kept in a pathogen-free environment in the animal house of the Instituto Nacional de Ciencias Médicas y Nutricion Salvador Zubiran (INCMNSZ). All experiments were conducted according to the ethical guidelines for animal handling required by the INCMNSZ.

The mouse mammary tumor cell line D2F2/E2 was cloned from a spontaneous mammary tumor that arose in the BALB/c hyperplastic alveolar nodule (HAN) line D2 was provided by Dr. Wei-Zen Wei (Wayne State University, Detroit, MI) [20, 21].

### Experimental model of allergic airway inflammation and breast cancer in BALB/c mice

After the induction of allergic airway inflammation as previously described [22], we inoculated 1 × 10^6^ D2F2/E2 cells into the right hind limbs of the mice via the subcutaneous (s.c.) route on day 11. The mice were divided into four experimental groups (6–8 mice per group). Group 1 was treated with OVA and inoculated with tumor cells. Group 2 was treated with saline solution (SS) and inoculated with tumor cells. Group 3 was treated with SS alone, and group 4 was treated with OVA alone. Once the tumor was palpable, it was measured weekly, and the tumor volume was calculated as 4/3*π* (½ smaller diameter)^2^ (½ larger diameter) = tumor volume (in mm^3^).

For the TGF-β activity inhibition assay, four intratumoral (I.T) inoculations of SB431542 (0.05 mg/kg) were performed on days 45, 47, 49 and 51 in the experimental model. The mice were killed on day 54. For the administration of TGF-β, three intratumoral inoculations of recombinant TGF-β (10 ng/ml) were performed on days 47, 49 and 51 in the experimental model. The mice were killed on day 54.

### Immunohistochemistry and digital pathology analysis

The expression levels of TGF-β, PCNA, TβRI, TβRII, phospho-Smad2 and phospho-Smad4 were analyzed in 4-µ tumor slices by immunohistochemistry using specific antibodies as previously described [23]. Slides were scanned to obtain electronic files. The immunohistochemical stains were digitally analyzed using the Aperio CS (San Diego, CA, USA) digital pathology equipment.

### Detection of apoptosis by terminal deoxynucleotidyl transferase dUTP nick end labeling (TUNEL) assay

DNA fragmentation in tumor tissue samples or tumor cell lines was evaluated by the TUNEL assay using an In Situ Cell Death Detection Kit (HRP) (Roche Applied Science, Mannheim, Germany) following the manufacturer’s instructions.

### TGF-β concentration determination by enzyme-linked immunosorbent assay (ELISA)

Cytokine concentrations were measured in individual serum samples from each experimental group with a commercial TGF-β ELISA kit following the manufacturer’s instructions (R&D Systems, Minneapolis, MN, USA).

### Proliferation assays

D2F2/E2 cells were labeled using the CellTrace Violet (CTV) Proliferation Kit (Molecular Probes, Eugene, OR, USA) according to the manufacturer’s instructions. Briefly, 3 × 10^6^ cells were labeled with CTV solution for 20 min at 37 °C. RPMI was added to stop the labeling reaction. After washing, the cells were cultured at a density of 1 × 10^5^ cells/ml either in the presence or in the absence of TGF-β (1 ng/ml) for 48 h at 37 °C. After culturing, the cells were harvested, washed and analyzed using a FACSCanto II flow cytometer (BD), and the resulting data were processed using FlowJo (Treestar, Inc.).

### Statistical analysis

Data were analyzed with the Prisma^®^ package and are expressed as the mean ± SD. Statistical comparisons were performed by ANOVA (analysis of variance) to determine group differences. Significant differences between two groups were determined using Student’s *t* test, and significant differences between three or more groups were determined with ANOVA and Tukey’s test or with the Bonferroni’s posttest. A value of *p* ≤ 0.05 was considered significant.

## Results

### Allergic airway inflammation inhibits cancer progression in an experimental mice model

**Fig. 1:**
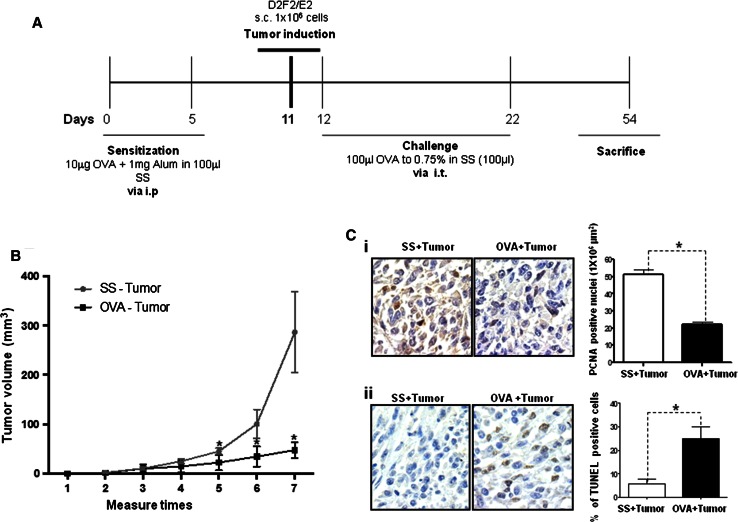
Allergic airway inflammation inhibits tumor growth. **a** Murine model of pulmonary allergic inflammation and tumor induction. Female BALB/c mice were sensitized and challenged with the indicated concentrations of OVA at the indicated time points. Tumors were induced by inoculation with the syngeneic breast cancer cell line D2F2/E2 at the indicated time point. At the end of the experiment, the mice were killed by exsanguination. *Alum* aluminum hydroxide, *SS* saline solution, *s.c.* subcutaneous, *i.p.* intraperitoneal, *i.t.* intratracheal. The different groups included SS, OVA, SS + tumor and OVA + tumor. **b** Tumor growth was evaluated weekly in the OVA + tumor and SS + tumor groups. The results are expressed as the means and standard deviations of three independent experiments. **p = 0.001 (Student’s *t* test). **c** Tumor samples from the OVA + tumor and SS + tumor groups were analyzed to determine the expression of PCNA by immunohistochemistry (**i**), and apoptosis was evaluated using an in situ TUNEL assay (**ii**). Magnification = ×40. PCNA, *p = 0.05 (Student’s *t* test). TUNEL, *p = 0.05 (Mann–Whitney *U* test)

To evaluate whether the immunologic conditions of allergic inflammation affect tumor progression, we used a mice model of allergic airway inflammation that was previously established by our research group [23, 24]. Murine breast cancer cells were also implanted in the experimental mice as described in the scheme shown in Fig. 1a. Figure 1b shows a significant decrease (*p* = 0.001) in the mean tumor volume in the allergy-with-tumor group (OVA + tumor) compared with the non-allergy-with-tumor group (SS + tumor).

An analysis of cell proliferation by immunohistochemical staining for PCNA [Fig. 1c(i)] revealed a significantly lower number of dividing cells in the OVA + tumor group compared with the SS + tumor group (*p* = 0.05). In contrast, the in situ TUNEL analysis demonstrated that the proportion of apoptotic cells in the OVA + tumor group was significantly higher (*p* = 0.04) than that in the SS + tumor group [Fig. 1c(ii)], suggesting that the inflammatory conditions of the allergic mice reduces tumor growth by inhibiting proliferation and inducing apoptotic cell death.

### Allergic airway inflammation induces the overexpression of TGF-β in both, serum and tumor tissue

**Fig. 2:**
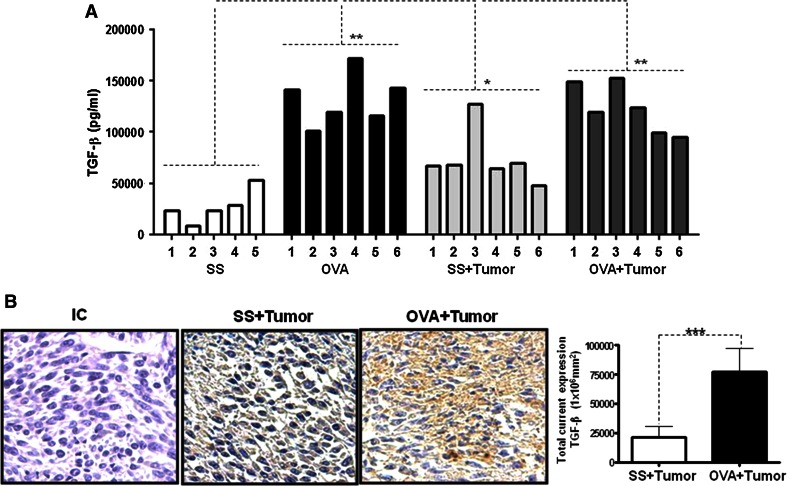
Allergic airway inflammation induces TGF-β overexpression. The level of systemic TGF-β was evaluated in sera from the SS, OVA, SS + tumor and OVA + tumor groups by quantitative ELISA (**a**). The assays were performed in triplicate using individual serum samples from each group in three independent experiments. The *error bars* indicate the standard error of the mean (**p = 0.001 SS vs. OVA, ***p = 0.0001 SS + tumor vs. OVA + tumor). The intracellular expression of TGF-β was evaluated in tumor samples from SS + tumor and OVA + tumor mice by immunohistochemistry (**b**). The antibody specificity was verified using an isotype control (IC) antibody. The expression density was evaluated by digital pathology using the Aperio CS system (*right panel*) (*p = 0.01)

To evaluate the mechanism by which the tumor volume decreases, we evaluated multiple cytokines using CBAs (cytometric bead array) (data not shown) and TGF-β expression by ELISA. Interestingly, TGF-β was the most overexpressed cytokine in the OVA + tumor mice in our experimental model. Figure 2a shows the concentration of TGF-β in the mice sera. A significant increase in the TGF-β concentration was detected in the OVA + tumor group compared with the SS + tumor group (*p* = 0.0001). The group of mice that received only OVA also exhibited a significant increase in TGF-β compared with the control group (SS) (*p* = 0.001). Interestingly, we also found an increased TGF-β level in the tumor tissue by immunohistochemical analysis. Figure 2b (left panel) shows more intense TGF-β staining in the OVA + tumor group compared with the SS + tumor group. Moreover, the quantification of the intracellular TGF-β expression by digital pathology revealed a significant increase in expression in the allergic mice (Fig. 2b, right panel) (*p* = 0.01). We also investigated the expression of type I (TβRI) and type II (TβRII) TGF-β receptors in the tumor tissue. As shown in Supplementary Figure 1, the tissue from the OVA + tumor mice expressed higher levels of both receptor types than the tumors from the SS + tumor mice.

These results clearly indicate that allergic inflammation induces not only the overexpression of systemic TGF-β but also stimulates the intracellular production of TGF-β in tumor cells.

### Inhibition of TGF-β activity induces tumor progression by increasing cell proliferation, and inhibiting apoptosis

**Fig. 3:**
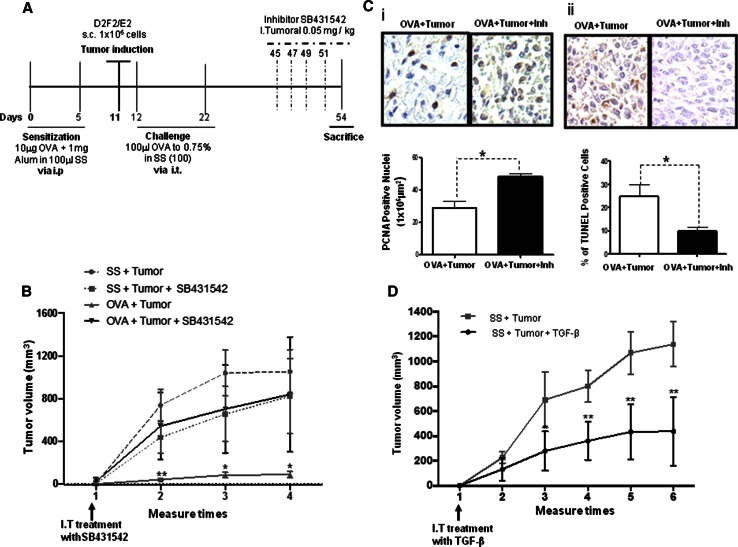
Inhibition of TGF-β activity induces tumor growth. **a** Experimental model for the inhibition of TβRI. **b** Tumor volume in the OVA + tumor + Inh group compared with the OVA + tumor group after intratumoral (I.T.) treatment with a TGF-β activity inhibitor (SB431542, 0.05 mg/kg) **p = 0.01. **c** Representative micrographs of PCNA immunohistochemistry (**i**) in tumor tissue after treatment of OVA + tumor + Inh and OVA + tumor mice with the TGF-β activity inhibitor (as in **b**) (*p = 0.007, Student’s *t* test with Bonferroni correction). **ii** The TUNEL assay in tumor tissue of OVA + tumor + Inh and OVA + tumor mice treated as in **b** (*p = 0.05, Student’s *t* test with Bonferroni correction). **d** Tumor volume in the SS + tumor mice either untreated or after intratumoral (I.T.) treatment with recombinant TGF-β (10 ng/ml) (**p = 0.01, Student’s *t* test with Bonferroni correction)

To demonstrate that the inhibition of tumor growth is mediated by TGF-β, we blocked the activity of this cytokine using a specific inhibitor of TβRI (SB431542) in vivo. Figure 3a shows a scheme of the inhibition process. To confirm the effect of the TβRI inhibitor, we evaluated the phosphorylation of Smad2 and Smad4, which are downstream targets of the receptor, by immunohistochemistry in tumor samples from allergic mice and allergic mice treated with the inhibitor. Supplementary Figure 2 shows representative micrographs of the Smad2 and Smad4 staining. As expected, phosphorylated Smad2 and Smad4 were detected in the untreated tumors (OVA + tumor). In contrast, the phosphorylation of both Smad2 and Smad4 was significantly reduced in the tumors treated with the inhibitor (OVA + tumor + Inh), indicating that blocking TβRI inhibits the TGF-β signaling cascade. Thus, we proceeded to evaluate the effect of the inhibitor on tumor growth. As shown in Fig. 3b, treatment with the inhibitor led to a significant increase in the tumor volume in the OVA + tumor + Inh animals compared with the tumor volume in the OVA + tumor animals (*p* = 0.01). Subsequently, we analyzed the effect of inhibiting TGF-β signaling on cell proliferation and apoptosis. As shown in Fig. 3e(i), cell proliferation, as demonstrated by the expression of PCNA, was significantly increased in the OVA + tumor + Inh group as compared with the OVA + tumor group (*p* = 0.007). In contrast, a significant decrease in the percentage of apoptotic cells was observed in the OVA + tumor + Inh group [Fig. 3e(ii)] (*p* = 0.05).

Next, we analyzed the direct effect of recombinant TGF-β on tumor growth. Interestingly, as shown in Fig. 3d, the administration of this cytokine to the group of mice with tumors and without allergy (SS + tumor + TGF-β) significantly decreased tumor growth compared with the tumor growth in the untreated group (SS + tumor).

These observations support the hypothesis that TGF-B is a negative regulator of tumor progression.

### Exogenous TGF-β inhibits proliferation and augments cell apoptosis

**Fig. 4:**
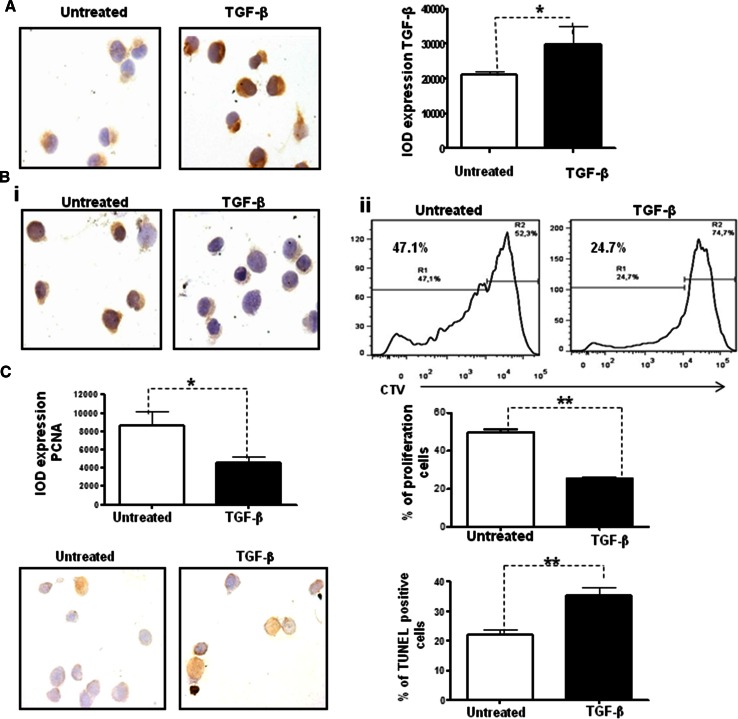
Exogenous TGF-β inhibits cell proliferation and augments cell apoptosis in vitro. D2F2/E2 cells were untreated or treated with recombinant TGF-β (1 ng/ml) for 48 h. **a** Representative immunocytochemistry micrographs and quantification of TGF-β (*p = 0.02). **b** Representative immunocytochemistry micrographs and quantification of PCNA (**i**) (magnification = ×100) (*p = 0.01). CTV staining and flow cytometry analysis for the evaluation of cell proliferation (**ii**) (**p = 0.01). **c** Representative micrographs and quantification of a TUNEL assay of D2F2/E2 cells either untreated or after exogenous TGF-β treatment (1 ng/ml for 48 h) (magnification = ×100) (**p = 0.02)

To further analyze the effect of exogenous TGF-β on tumor cells, we incubated D2F2/E2 cells in the presence of recombinant TGF-β for 48 h. We first evaluated the expression of intracellular TGF-β by immunocytochemistry. As shown in Fig. 4a, stimulation with exogenous TGF-β induced a significant increase in the expression of intracellular TGF-β (*p* = 0.01). Consistent with this finding, the administration of exogenous TGF-β significantly inhibited cell proliferation, as demonstrated by the PCNA expression [Fig. 4b(i)] (*p* = 0.01) and the CTV assay conducted using flow cytometry [Fig. 4b(ii)] (*p* = 0.03). Furthermore, the administration of exogenous TGF-β induced a significant increase in apoptosis, as demonstrated by the in situ TUNEL assay (Fig. 4c).

Taken together, our results revealed that exogenous TGF-β induces the overexpression of intracellular TGF-β, which in turn inhibits proliferation and increases the apoptosis of tumor cells.

## Discussion

There is conflicting evidence regarding the association between allergic disease and cancer. Although these two processes have been studied together in a single experimental model, currently, no studies have evaluated the functional effects of pulmonary allergic inflammation, which is characteristic of asthma, on breast cancer progression.

In a former study using mice with OVA-triggered pulmonary allergic inflammation, and urethane-induced lung cancer, the authors found that the allergic status had no influence on the development of the neoplasia [15]. However, it was recently shown that blocking TGF-β expression in mice with urethane-induced lung cancer leads to a significant increase in tumor progression [16]. This finding emphasizes the importance of TGF-β in suppressing tumor progression, at least in the case of chemically induced lung cancer. However, the above-mentioned studies did not provide information on the progression of tumors during an allergic response. In the present study, we used a well-established murine allergic airway inflammation model [23] to demonstrate the influence of this type of response, which is characterized by an increase in TGF-β, on breast cancer progression. We initially compared the tumor volumes in the allergic mice with those in the nonallergic mice. Our results demonstrated that the allergy significantly inhibited tumor growth. This study is the first to demonstrate the effects of pulmonary allergic inflammation, which is characteristic of asthma, on breast cancer progression.

We were interested in elucidating the mechanism underlying tumor growth inhibition. We analyzed the role of TGF-β during this process because there is compelling evidence suggesting that it acts as a potential inhibitor of tumor growth in an experimental model of lung cancer [16]. Our results showed that the expression of this cytokine increased in the serum and tumor tissue from the OVA + tumor group. The TGF-β level is known to be elevated in allergic diseases, and this elevation is correlated with disease severity [9–11, 14, 15], which explains the high TGF-β concentration in allergic mice. However, the finding that TGF-β was only increased in the group of allergic mice with tumors (OVA + tumor) and not in the nonallergic mice with tumors (SS + tumor) is interesting because it strongly suggests that TGF-β may function as an inhibitor of tumor growth in our experimental model. This possibility is consistent with the data reported by Luo et al. [16], who demonstrated the antitumoral effects of TGF-β activity, and by Novitskiy et al. [25], who reported that the loss of the TGF-β receptor (and thus the activity of the cytokine) promoted breast cancer progression.

It has been reported that TGF-β induces apoptosis in tumor cells in vitro [26, 27]. Our results demonstrated that the level of TGF-β in the serum and tumor tissue was higher in allergic mice than nonallergic mice. Allergic inflammation reactions are regulated by a number of cytokines and growth factors. Vascular endothelial growth factor (VEGF) is an important mediator of chronic inflammatory airway disease [28]. Interestingly, a recent report showed that TGF-β1 is able to induce endothelial cell apoptosis by shifting VEGF signaling from the prosurvival p38β MAPK (mitogen-activated protein kinase) isoform to the proapototic p38α isoform [29]. This finding is interesting because VEGF is another cytokine that is known to contribute to the pathogenesis of pulmonary allergic inflammation [28–30]. It would be pertinent to evaluate the role of this pathway in apoptosis induction in our model and to determine whether it can also inhibit tumor progression. Our results are consistent with those of Pinto et al. [31], which suggested that a concomitant allergic condition (e.g., a food allergy) reduces tumor progression by increasing tumor cell apoptosis that is accompanied by a reduced area of necrosis at the tumor site. Indeed, these findings suggest that an increase in apoptosis is a possible mechanism for the lower incidence of cancer observed in allergic individuals.

To demonstrate that the overexpression of TGF-β generated in the allergic airway inflammation model was partially responsible for the increased apoptosis and the inhibition in cell proliferation that resulted in the inhibition of tumor progression, we performed an in vitro study in which we treated tumor cells with exogenous TGF-β. Remarkably, we demonstrated that administration of exogenous TGF-β produced an increment on the level of intracellular TGF-β expressed by tumor cells, inhibiting proliferation and augmenting apoptosis. These results are consistent with the in vivo studies and strongly suggest that the overexpression of this cytokine in the tumor microenvironment is at least partially responsible for the inhibition of tumor progression.

TGF-β plays a major role in cancer by suppressing tumor growth during the early phase of neoplasia and by promoting tumor progression and metastasis in later phases [32]. Thus, many malignant tumors produce large amounts of TGF-β but are resistant to its growth-inhibitory effects.

In the context of the immune response, TGF-β is a key factor that induces regulatory T cells that exert powerful and diverse immunosuppressive effects. However, not all of the effects of TGF-β are suppressive. In combination with IL-6, TGF-β induces Th17 differentiation. These effects have not only been linked to inflammation and autoimmunity but have also been linked to a protective role in certain types of cancer [33]. This capacity of TGF-β to induce either immunosuppressive or inflammatory events is dependent on the cytokine microenvironment, which must be considered when analyzing its role in a particular disease [34–36]. As we previously mentioned, allergic asthma has been considered to be a Th2-biased disease [4–7]. However, a Th17-biased response has also been observed in patients who exhibit allergic inflammation [37, 38], particularly in those with severe asthma who respond poorly to steroids, in whom inflammatory cellular infiltration in the airway is primarily due to CD4^+^ Th17 cells [39]. Therefore, it is possible that in our experimental model, under allergic conditions, TGF-β could also play a protective role due the induction of a Th17 cell-mediated response.

**Fig. 5:**
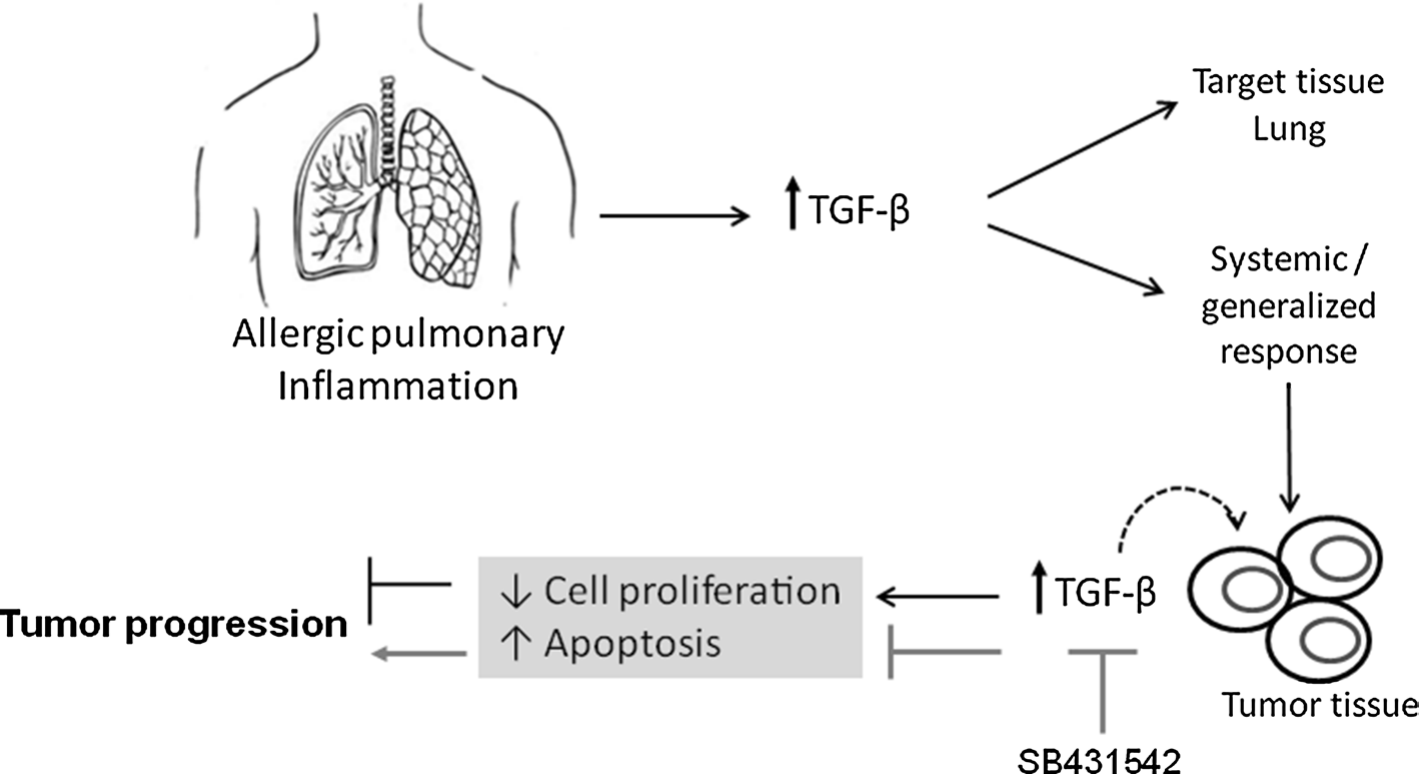
A model of tumor progression inhibition mediated by TGF-β during pulmonary allergic inflammation. There is an inverse relationship between pulmonary allergic inflammation and tumor progression. The mechanism by which tumor progression is inhibited in our model is mediated by TGF-β, which is overexpressed during allergic airway inflammation. In the tumor microenvironment, TGF-β leads to a further increase in the level of intracellular TGF-β and its receptors, which in turn inhibits proliferation and increases tumor cell apoptosis. When the TGF-β activity is inhibited with SB431542, cell proliferation increases, apoptosis is inhibited, and tumor progression ensues

In conclusion, our results are the first to establish an inverse relationship between allergic airway inflammation and tumor progression in which TGF-β overexpression plays a direct, relevant role. We also show that the inhibition of tumor progression in our model is dependent on TGF-β, which is overexpressed in allergic airway inflammation and induces tumor cell apoptosis in addition to inhibiting proliferation (see the proposed model in Fig. 5). Taken together, the results obtained in this study indicate that TGF-β is a potential target for antitumor therapy.

## Electronic supplementary material

Supplementary material 1 (PDF 226 kb)

## Abbreviations

Alum: Aluminum hydroxide
CTV: CellTrace Violet
ELISA: Enzyme-linked immunosorbent assay
HAN: Hyperplastic alveolar nodule
i.p.: Intraperitoneal
i.t.: Intratracheal
I.T.: Intratumoral
MAPK: Mitogen-activated protein kinase
OVA: Ovalbumin
PCNA: Proliferating cell nuclear antigen
HRP: Horse-radish peroxidase
pSMAD-2: Phosphorylated SMAD homologue 2
pSMAD-4: Phosphorylated SMAD homologue 4
s.c.: Subcutaneous
SMAD: Intracellular signal transduction proteins for transforming growth factor β receptors
SS: Saline solution
TGF-β: Transforming growth factor β
Th17: Interleukin (IL)-17-producing T helper cells
Th1: T helper type 1 cells
Th2: T helper type 2 cells
TUNEL: Terminal deoxynucleotidyl transferase dUTP nick end labeling
TβRII: TGF-β receptor II
TβRI: TGF-β receptor I
VEGF: Vascular endothelial growth factor
