# Supplementary material for: Inhibition of tumor progression during allergic airway inflammation in a murine model: significant role of TGF-β
Source: Cancer Immunol Immunother. 2015 Jun 16;64(9):1205–14. doi: 10.1007/s00262-015-1722-4 (PMC4540764; doi:10.1007/s00262-015-1722-4)
Supplement: Supplementary file 1 — Supplementary material 1 (PDF 226 kb) [file 262_2015_1722_MOESM1_ESM.pdf]

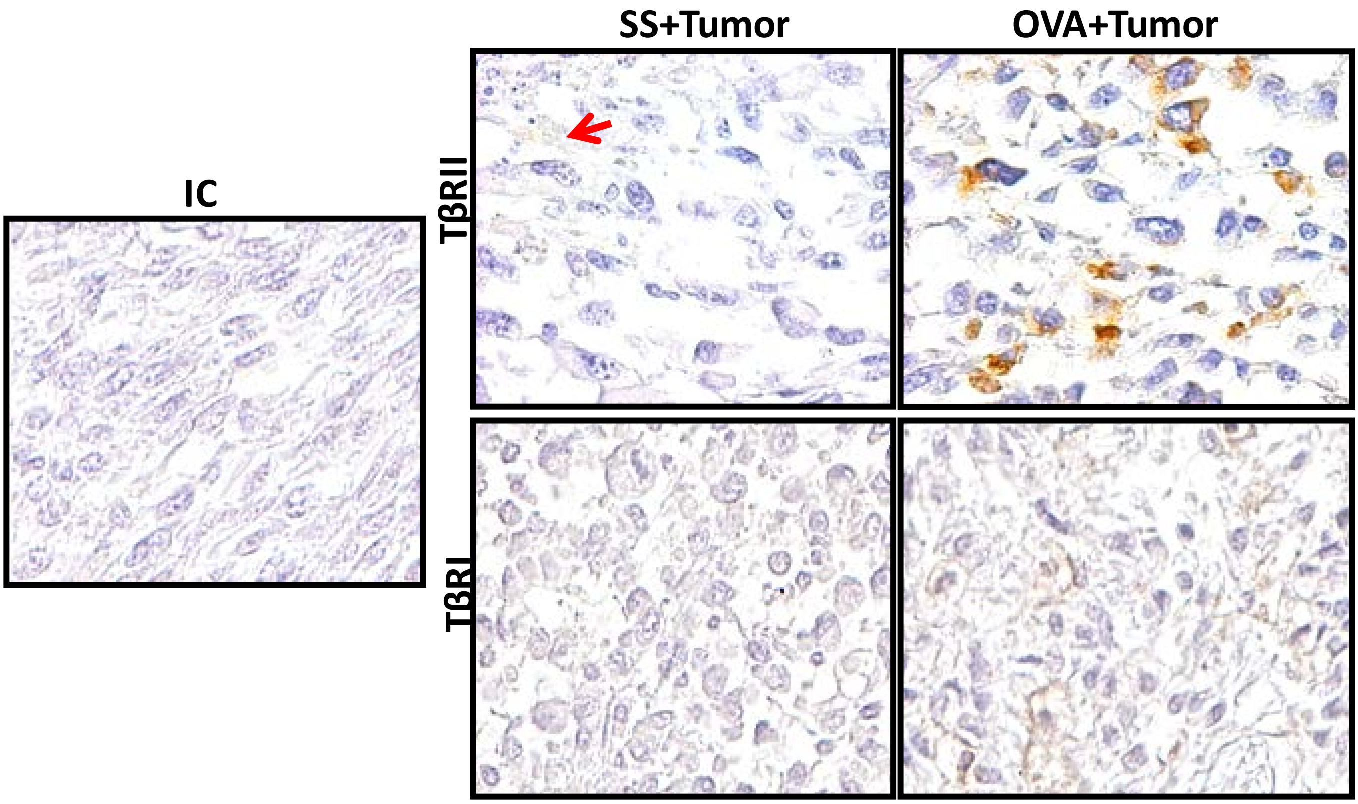

Supplementary Figure 1. Expression of the receptors TβRI And TβRII in a tumor tissue. The expression of the receptors TβRI and TβRII was evaluated by immunohistochemistry in tumor samples from SS+Tumor and OVA+Tumor mice. The antibody specificity was corroborated using an Isotype control (IC). The micrographs are representative of three independent experiments. Magnification=100X.

Cancer Immunology, Immunotherapy (submitted in 2014) – Belen Tirado-Rodríguez et al.

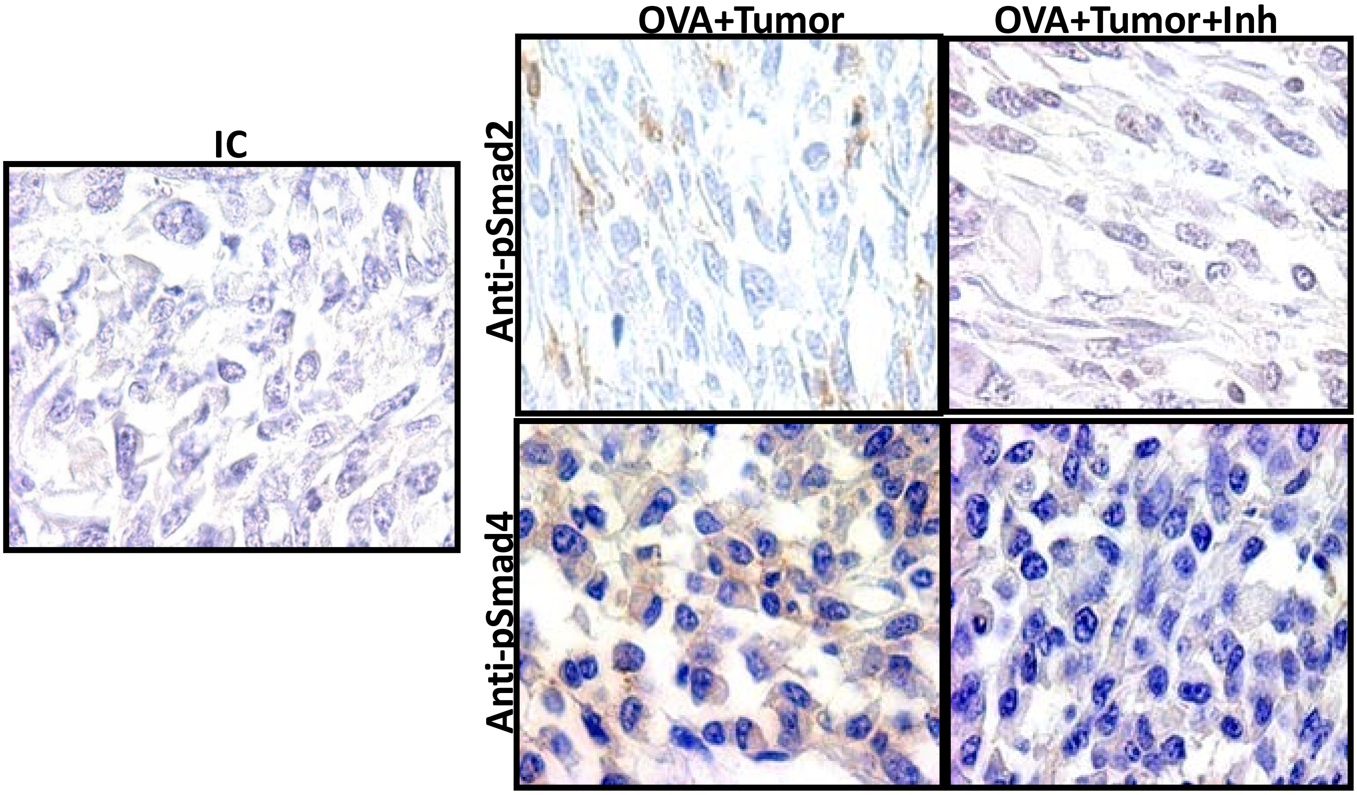

Supplementary Figure 2. Evaluation of the activity of TGF-β ihibitor. The phosphorylation of Smad2 (upper panel) and Smad4 (bottom panel) was examined by immunohistochemistry in tumor tissue; it is clear that in the OVA+Tumor group, the levels of phosphorylated Smad2 and Smad4 increased in the tumor tissue, whereas in the OVA+Tumor+Inh group, phosphorylated Smad2 and Smad4 were at basal levels. The antibody specificity was established with an IC that did not yield immunohistochemistry. Magnification=100X.

Cancer Immunology, Immunotherapy (submitted in 2014) – Belen Tirado-Rodríguez et al.
